# Prevalence, sociodemographic determinants and self-reported reasons for hysterectomy in India

**DOI:** 10.1186/s12978-019-0780-z

**Published:** 2019-08-02

**Authors:** Chander Shekhar, Balram Paswan, Abhishek Singh

**Affiliations:** 1 Department of Fertility Studies, International Institute for Population Sciences (IIPS), Mumbai, India; 2 Department of Population Policies and Programmes, International Institute for Population Sciences (IIPS), Mumbai, India; 3 Department of Public Health and Mortality Studies, International Institute for Population Sciences (IIPS), Mumbai, India

**Keywords:** Hysterectomy, Prevalence, Sociodemographic, Determinants, Self-reported reasons, India

## Abstract

**Background:**

Evidence of hysterectomy in India is limited mainly due to lack of information in large-scale nationally representative health surveys. In 2015–16, the fourth National Family Health Survey (NFHS-4) – a cross-sectional survey – collected for the first time direct information on hysterectomy and self-reported reasons for undergoing the procedure among women in the reproductive age group. This paper examines the prevalence and determinants of hysterectomy in India among women aged 30–49 years in 29 states and seven union territories (UTs) of India using the NFHS-4 dataset.

**Methods:**

Percentage weighted by sampling weights was used for estimating the prevalence of hysterectomy. The paper used crosstabulations and percentage distributions to estimate the prevalence of hysterectomy across different socioeconomic backgrounds and reasons for undergoing hysterectomy respectively. A multivariate binary logistic regression model was also used to find statistically significant determinants of hysterectomy.

**Results:**

In India as a whole, 6 % of women aged 30–49 years had undergone a hysterectomy. The percentage of women who had undergone the procedure was found to vary considerably across the states and the UTs (from a minimum of 2% in Lakshadweep to a maximum of 16% in Andhra Pradesh). A noticeable fact that emerged was that the majority of the hysterectomies were performed in the private sector except in the northeast region. Years of schooling, caste, religion, geographic region, place of residence, wealth quintiles, age, parity, age at first cohabitation, marital status, and body mass index of women were found to be the sociodemographic determinants statistically associated with hysterectomy in India. The reasons reported frequently for hysterectomy were excessive menstrual bleeding/pain (56%), followed by fibroids/cysts (20%).

**Conclusion:**

The percentage and likelihood of undergoing hysterectomy are relatively high among women from older age groups (45–49), those who reside in rural areas, those without schooling, those who are obese, those having high parity, those with a low age at first marriage, and those who reside in the eastern and southern parts of India. The policy implication of these findings is that the reproductive health program managers should ensure regular screening and timely treatment of the problems resulting in hysterectomy.

## Plain English summary

Hysterectomy is a surgery that involves partial or complete removal of the uterus of a woman. In India, nationally representative reliable statistics are rarely available on this important aspect of women’s health. The fourth round of the National Family and Health Survey (NFHS-4) canvassed, for the first time, a battery of direct questions on hysterectomy and the reasons for undergoing it.

This paper used the NFHS-4 dataset to estimate the prevalence of hysterectomy in the states and the Union Territories in India by different socioeconomic backgrounds of women. It also shows the statistical significance and likelihood of women from different socioeconomic backgrounds undergoing a hysterectomy.

Six percent of women in the age group 30–49 years have undergone a hysterectomy in India as a whole. Women who come from the rural areas, had no schooling, have a body mass index more than or equal to 25 kg/m2, belong to a high wealth quintile household, have higher parity, had a low age at marriage, and are from eastern and southern India are more likely to undergo a hysterectomy. Among the main reasons for hysterectomy reported by women aged 30–49 years are excessive menstrual bleeding/pain (56%), fibroids/cysts (20%), and uterine disorder, that is, rupture (14%) and uterine prolapse (8%).

Translating the prevalence of hysterectomy into absolute numbers for the year 2016, there were about 10 million hysterectomized women (aged 30–49 years) living in India. This huge number may further increase if reasons for hysterectomy are not diagnosed and treated effectively at an early stage. Policymakers and program managers must ensure timely and quality reproductive health services up to the primary healthcare level.

## Introduction

Hysterectomy is a surgical operation whereby the uterus of a woman is removed. There are different types of hysterectomy such as partial, total and radical hysterectomy. In case of partial hysterectomy, the upper part of the uterus is removed but the cervix is left in place. In total hysterectomy, the whole uterus, including the cervix, is removed. And in radical hysterectomy, the whole uterus, the tissue on the sides of the uterus, the cervix, and the top part of the vagina are removed. Radical hysterectomy is generally suggested to women in case of cancer.

Hysterectomy is primarily done to save women from uterus-related life-threatening problems and for a better and healthy life. It is done for different gynecological conditions that include uterine fibroids that cause pain, bleeding, or other problems; uterine prolapse; cancer of the uterus, cervix or ovaries; endometriosis; abnormal vaginal bleeding; chronic pelvic pain; or Adenomyosis. In case of noncancerous situations, it is considered only after all other treatment approaches have failed or have been exhausted [1].

A number of studies have looked at the socioeconomic, demographic, and residence related correlates of hysterectomy in different settings, including India [2–8]. A cohort study examined the risk factors for peripartum hysterectomy. Using data from 193 hospitals from 21 countries from Africa, Asia, Europe, and the Americas, the study concluded that placenta praevia/accreta is associated with a higher risk of peripartum hysterectomy. Women in Asia had a higher hysterectomy incidence (7%) than women in Africa (5%) (adjusted odds ratio: 1.2, 95%confidence interval (CI): 0.9–1.7). The other risk factors the study found were advanced maternal age, caesarean section, and giving multiple births in Asia [7].

A few studies have also examined other risk factors for hysterectomy. A study by Whiteman et al. (2006) reported that primary caesarean deliveries, repeat caesarean deliveries, vaginal birth after caesarean, and multiple births were independently associated with an increased risk for peripartum hysterectomy [9]. Dharmalingam, Pool and Dickson (2000), using nationally representative data from New Zealand, found that older women, women who experienced foetal loss, women of higher parity, married women, women who used intrauterine devices (IUDs) and pills, and women with tubal sterilization were at a high risk for hysterectomy [3]. In rural China, women at a higher age (40 years and above), those with a higher body mass index, and those who had experienced loss of a child previously were at a higher risk for hysterectomy [10].

The knowledge regarding hysterectomies in India is limited partly due to lack of information in large-scale national representative surveys. Two mixed method studies in a low income setting in Gujarat in India found that women with low income, those at a higher age, rural women, married women, and women with more surviving children were at a higher risk for hysterectomy. The study revealed that the mean age at hysterectomy was 36 years and that the majority of the women had used private health facilities for hysterectomies [5, 6].

A study in three villages of Panchkula district in Haryana revealed that the prevalence of hysterectomy was more in women above the age of 35 years. Excessive menstrual bleeding was the main indication for hysterectomy (52/70; 74%); uterine prolapse and fibroids were the other indications [11]. In an urban setting of district Moradabad in Uttar Pradesh, a study found that women who had their first pregnancy before the age of 20 years had a 2.5 times higher chance of undergoing a hysterectomy than those who first became pregnant at age 20 years or later [4].

A study was recently carried out using secondary data from the fourth round of the district level household survey (2012–13) done among married women aged 15–49 years in 21 states and UTs [8]. It found that the likelihood of hysterectomy was 1.9 times higher among women belonging to households having a health insurance, which raised suspicions about the misuse of the procedure. It found that the prevalence of hysterectomy across 21 states and UTs ranged from 0.2% to 6.3%. A major limitation of this study was the national representation since the 21 states covered in the survey represented only about 52% of the total married women aged 15–49 years in India. The other major limitations included lack of information on the age at hysterectomy, type of facility where the procedure was done, and reasons for hysterectomy.

A study by Radha et al. (2015) in Tirupati city of Andhra Pradesh found that the common indications for hysterectomy were fibroids (45%), menorrhagia (31%), prolapse (10%), endometrial hyperplasia (5%), cervical dysplasia (3%), and chronic pelvic inflammatory disease (5%) [12]. In another recent study, pelvic organ prolapse was the most common indication for hysterectomy, while uterine fibroids, dysfunctional uterine bleeding, ovarian mass, chronic pelvic inflammatory disease, and cervical intraepithelial neoplasia were the other indications [13]. Pandey et al. (2014) did a hospital based study and found that the most common indication for hysterectomy was symptomatic uterine fibroid (40%), followed by prolapse (16%), and that the overall complication rate was 8.5% [14].

Uikey, Wankhede and Tajne (2018) found that the commonest indication for hysterectomy was fibroid uterus (65.33%) in Maharashtra state of India. They concluded that in a developing country like India with poor healthcare resources, non-descent vaginal hysterectomy offers a distinctive advantage over abdominal and laparoscopic assisted vaginal hysterectomy and should be the route of choice for benign uterine conditions [15].

Some researchers and activists have questioned the unnecessary hysterectomies being carried out in some parts of India for commercial gains rather than medical necessity, and at a much younger age of women in states like Andhra Pradesh [16–19]. There has also been considerable debate concerning the efficacy of elective hysterectomy as reproductive health problems of women do not end there [20]. Once a hysterectomy has been done, many health complications start such as: i) early onset of menopause, ii) higher risk of cardiovascular disease, iii) higher risk of stroke, iv) urinary incontinence, v) loss of sexual desire, and vi) other health problems [5, 6].

A study done by Verma, Singh and Kulshrestha (2016), among patients in a north Indian city, justified hysterectomy medically only when the histopathological examination (HPE) report is compatible with the preoperative diagnosis and recommended that the HPE should be done and analysed for all hysterectomy specimens. It found that uterovaginal prolapse (38%) was the most common preoperative indication, while fibroid uterus (26%) was the commonest indication for abdominal hysterectomy. Other common indications were dysfunctional uterine bleeding (10%) and ovarian mass (8%) [21].

Most of the literature available on hysterectomies is from studies conducted in the developed world or from clinic based samples. The literature available with regard to India is limited in nature and scope. To our knowledge, a population based study that can cover India as a whole has not yet been conducted on a large-scale nationally representative dataset. It is important to mention here that the National Family and Health Survey in its fourth round (NFHS-4) collected, for the first time, direct information on hysterectomy from women in the reproductive age group. Having noted the gaps in the existing literature on hysterectomy in India and given the availability of a recent large-scale population based nationally representative dataset, the present study investigated the prevalence and determinants of hysterectomy in women aged 30–49 years in India. We restricted our analysis to women aged 30–49 years because previous studies have shown that women of higher age are at a high risk of hysterectomy [2, 3, 5, 6, 10, 22].

This paper seeks answers to the following questions: (i) what is the prevalence of hysterectomy in different sociodemographic subgroups of Indian women aged 30–49? ii) do women from different sociodemographic subgroups carry significantly different risks to undergo hysterectomy? and (iii) what are the variations across states/UTs in self-reported reasons for seeking hysterectomy? Therefore, the specific objectives of this paper are: i) to understand the prevalence of hysterectomy among women aged 30–49 years at the national, state, UT and regional levels in India, ii) to examine the sociodemographic determinants of hysterectomy, and iii) to examine the reasons reported by women for hysterectomy.

## Materials and methods

### Study setting and participants

The dataset used in this paper has been taken from the Demographic and Health Survey, popularly known as the National Family and Health Survey (NFHS-4), conducted during 2015–16. The survey was conducted by the International Institute for Population Sciences (IIPS), Mumbai, under the aegis of the Ministry of Health & Family Welfare, Government of India. The main objectives of the survey were to provide the estimates of fertility, mortality, family planning, maternal and child health indicators, domestic violence, women’s autonomy, and non-communicable diseases and associated risk factors. The NFHS-4 collected information from 699,686 women aged 15–49 years, including 340,127 women aged 30–49 years, from 29 states and seven UTs of India [23].

The survey adopted a two-stage design in both rural and urban areas. In the rural areas, villages were selected in the first stage using the probability proportional to size scheme (PPS). Twenty-two households were chosen from the earmarked villages using systematic sampling in the second stage. In the urban areas, census enumeration blocks (CEBs) were selected in the first stage using PPS, and 22 households were selected in the second stage using systematic sampling. Women (15–49) residing in the selected households were interviewed with a 97% response rate. Only women in the age bracket 30–49 years were considered as the unit of analysis. The data analysis is secondary and remains mainly descriptive in nature.

As per the survey protocol of NFHS-4, ethical approval was sought from the IIPS Institutional Ethical Review Board with certificate ref. no./IRB/NFHS-4/01_1/2015.

### Outcome and independent variables

The outcome variable used in the present analysis is hysterectomy. The NFHS-4 asked women various questions related to hysterectomy. The first question asked was: “When did your last menstrual period start?” Among the several answers to this question, one of the options was “Has had hysterectomy”. The direct question on hysterectomy canvassed was “Some women undergo an operation to remove the uterus. Have you undergone such an operation?” If the answer was yes, women were asked further questions regarding the timing and place of and the reason for the hysterectomy.

The independent variables, their categorization and their definitions are given in the following table.

**Table:** 

Independent variable	Definition	Categories
Age	Biological age of women respondent	30–34;35–39;40–44;45–49
Women’s education	Educational attainment of women depending on years of schooling	No Schooling (0 years of schooling); Primary complete (5 years of schooling); Secondary complete (6–12 years of schooling); Higher (13 years and above schooling)
Caste/Tribe	Scheduled Caste/Tribe, Other Backward Classes as defined in the Indian constitution for the socially and economically deprived sections of the society	Scheduled caste; Scheduled tribe; Other Backward Class; Others (does not belong to any of the above three groups)
Religion	Religion in which the respondent believes	Hindu; Muslim (Islam); Christian; Sikh; Buddhist/neo-Buddhist; Other (Other than the above four religions)
Residence	Place where the respondent usually lives	Urban; Rural
Wealth Index	Household wealth index created by using scores of possession of certain goods and assets and classified in quintiles. Score moving from lowest to highest means household moving from poor to rich category.	Lowest; Second; Middle; Fourth; Highest
Body Mass Index (BMI) of women	BMI is Weight (in kilogram) of a woman divided by the square of height (in meters) of women;	BMI < 25 kg/m^2^;BMI > =25Kg/m^2^; BMI Not Available (could not be measured)
Marital Status	Current marital status of women; Others includes divorce, separated, and living together without marriage	Currently Married; Widow; Others
Age at first cohabitation	Age (in years) at which woman started living with spouse	Less than 15; 15–19; 20 and above
Parity	Total no. of children ever born to women	0; 1–2; 3 and above
Region	Region comprising a group of states, depending upon the geographical region and the sociocultural milieu these states fall in^1^	North; Central; East; Northeast; West; South
Time since hysterectomy (in years)	No. of years gone by after a woman underwent a hysterectomy	Less than 3; 3–5; 6–10; 10 and above years
Place of hysterectomy	Place where a woman underwent a hysterectomy; it is divided into two groups: public and private health facilities*	Public health facilities; Private health facilities

*India’s health system can be easily divided into public and private health systems. In general, both public and private health systems can be further categorised into a three-tier health care system (primary, secondary, tertiary). Primary and secondary public health care system is predominantly located in rural areas. On the other hand, private and tertiary public health care system is mainly located in urban areas. The socioeconomically poor and those belonging to the rural strata of the Indian society are primarily dependent on the public health system in seeking all types of healthcare services. Sometimes, inaccessibility, inefficiency, and quality issues with the public healthcare system force people to go to the private providers, pushing them into poverty due to the necessity of having to pay a heavy out-of-pocket expenditure even for basic primary healthcare needs

Crosstabulation, Chi-square, and a multivariate logistic regression (Backward Stepwise (Likelihood Ratio)) were used to analyse the data. The Omnibus Tests of Model Coefficients is used to test whether the new model got improved over the base model. The test uses Chi-square values to test weather -2Log-likelihood has significantly improved from the base to the final mode. To test the difference in the estimated median for the selected backgrounds, the K-independent sample non-parametric test was used. Appropriate sampling weights were used in the calculation of percentages, percent distributions, and prevalence. The details of the sampling weights are given in the national report [23]. The analysis was carried out in statistical package for social sciences (SPSS) 17.0. We also used geographical information system (GIS) to prepare a thematic map.

## Results

### Prevalence and geographical pattern of hysterectomy in India

The proportion of women who have undergone a hysterectomy is not low in India, as is evident from the latest empirical data of the NFHS-4. The proportion of women who have undergone a hysterectomy was 6 % among women aged 30–49 (Table 1).

**Table 1:** Percentage of women (30–49) undergone hysterectomy by sociodemographic and geographical background in India, NFHS (2015–16)

	Total number of women	Number of women with hysterectomy	Percentage of women with hysterectomy	*P*-value (Chi-square)
Age				*p* < 0.000
30–34	96,769	2426	2.5	
35–39	90,890	4314	4.8	
40–44	71,970	6054	7.8	
45–49	74,497	7967	10.7	
Women’s education				*p* < 0.000
No schooling	1,40,926	10,515	7.5	
Primary complete	50,357	3490	6.9	
Secondary complete	1,19,795	5958	5.0	
Higher	29,048	798	2.7	
Caste/tribe				*p* < 0.000
Scheduled caste	67,159	3744	5.6	
Scheduled tribe	29,944	1346	4.5	
Other backward class	1,47,083	10,180	6.9	
Other	95,942	5492	5.7	
Religion				*p* < 0.000
Hindu	2,78,184	17,749	6.4	
Muslim	41,681	2003	4.8	
Christian	8887	516	5.8	
Sikh	6012	312	5.2	
Buddhist/Neo-Buddhist	3168	87	2.7	
Other	2195	94	4.3	
Residence				*p* < 0.000
Urban	1,22,950	6359	5.2	
Rural	2,17,176	14,402	6.6	
Wealth index				*p* < 0.000
Lowest	59,060	2685	4.5	
Second	63,654	3925	6.2	
Middle	67,611	4771	7.1	
Fourth	72,732	4966	6.8	
Highest	77,069	4414	5.7	
BMI of women^#^				*p* < 0.000
BMI < 25 kg/m^2^	2,31,322	12,457	5.4	
BMI > =25.0 kg/m^2^	1,00,871	7847	7.8	
BMI Not Available	7933	457	5.8	
Marital Status				*p* < 0.000
Currently Married	3,09,902	18,978	6.1	
Widow	19,999	1468	7.3	
Others	10,225	314	3.1	
Age at first cohabitation (years)				*p* < 0.000
Less than 15	49,767	4944	9.9	
15–19	1,54,380	9771	6.3	
20 and above	1,05,877	4272	4.0	
Not Applicable/available	30,102	1774	5.9	
Parity				*p* < 0.000
0	15,729	403	2.6	
1–2	1,45,988	8166	5.6	
3 and above	1,78,408	12,191	6.8	
State/union territory	Total number of women	Number of women with hysterectomy	Percentage of women with hysterectomy	P-value (Chi-square)
Region				*p* **<** 0.000
North	45,375	1955	4.3	
Central	73,527	3767	5.1	
East	73,111	4685	6.4	
Northeast	12,126	252	2.1	
West	50,723	3022	6.0	
South	85,265	7080	8.3	
India	3,40,127	20,761	6.1	

**Fig. 1:**
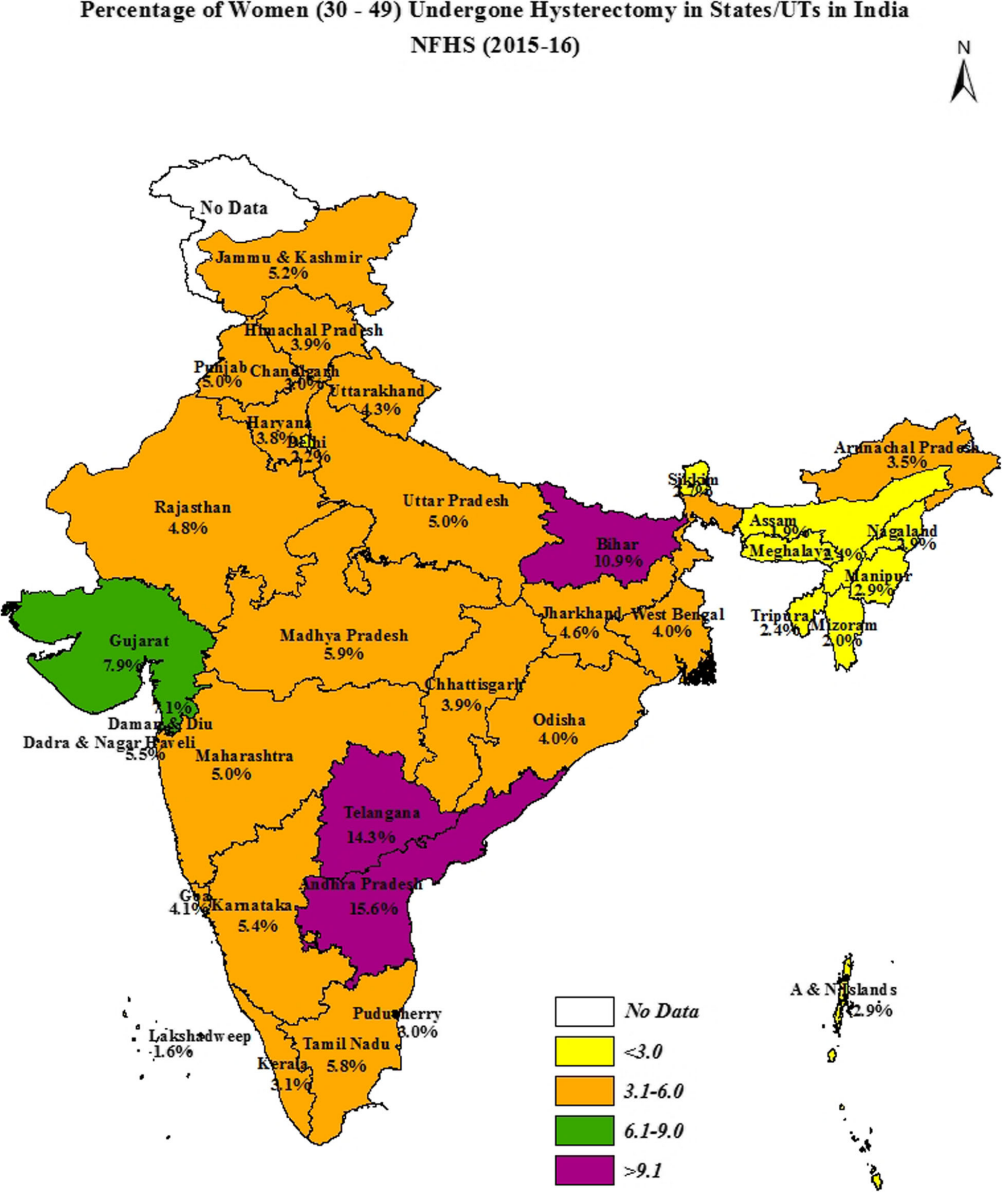
Prevalence of hysterectomy by state/UTs in India

Figure 1 shows that the percentage of hysterectomies carried out among women aged 30–49 was the highest in Andhra Pradesh, which at 16% is much higher than the national level of 6 %. The state of Telangana ranked second, where 14% women aged 30–49 had undergone hysterectomies, followed by Bihar (11%), Gujarat (8%), and Tamil Nadu (6%). Among UTs, Dadra & Nagar Haveli showed the highest prevalence of hysterectomy (7%), whereas Lakshadweep (2%) had the lowest prevalence. There is a considerable variation in the proportion of women aged 30–49 undergoing hysterectomy by different geographical regions in India.

### Contrasts in median age of hysterectomy in India

**Fig. 2:**
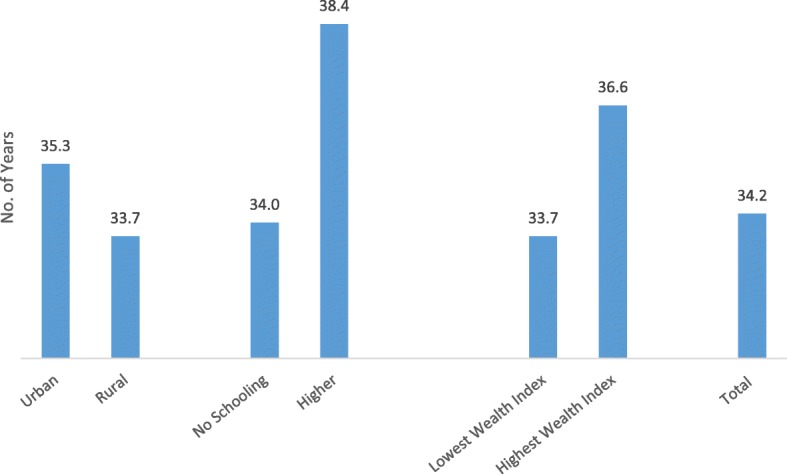
Socioeconomic contrasts in median age of hysterectomy in India, NFHS (2015–16)

Figure 2 provides the median age (in years) of women at hysterectomy in India and variations across residence, education, and wealth index. It shows that half of those who reported to have had a hysterectomy had undergone it by the age of 34 years among women aged 30–49 years. The median age at hysterectomy was about 2 years higher among urban women (35.3 years) than rural women (33.7 years). Women with no schooling had a hysterectomy at a younger median age (34 years) than women educated up to 12 or more years of schooling (38.4 years). A clear contrast appeared between women belonging to the lowest and the highest wealth quintile households, with the median age at hysterectomy of women belonging to the lowest quintile being 2.4 years younger than that of women from households in the highest wealth quintile. All the median ages for hysterectomy across residence, educational and household wealth index categories, shown in Fig. 2, were found significantly different at *p* < 0.000 when K-independent sample non-parametric test was applied.

### Socioeconomic differentials in hysterectomy in India

Table 1 presents the percentage of women aged 30–49, who have had a hysterectomy, by socioeconomic and demographic characteristics. A considerable variation by socioeconomic and demographic characteristics of women is observed in the risk of hysterectomy. The level of hysterectomy increases with an increase in age. About 3 % of women aged 30–34 reported having undergone a hysterectomy; this proportion increased to 5 % among women aged 35–49, 8 % among women aged 40–44, and to 11% among women aged 45–49. The risk of hysterectomy was higher among women with no schooling. Around 8 % of women with no schooling had a hysterectomy compared with only 3 % women with higher education. Seven per cent women aged 30–49 with “primary complete” level of education and 5 % women with “secondary complete” level of education had undergone a hysterectomy in the NFHS-4.

A higher proportion of other backward class women (7%) reported having undergone a hysterectomy compared with 6 % women belonging to other than scheduled castes, scheduled tribes, and other backward class. Among scheduled caste women, the prevalence was 6 %, and among scheduled tribe women, it was 5 %. The percentage of hysterectomized women was the highest among Hindu women (6%), followed by Christian women, and the lowest among Buddhist/Neo Buddhist women (3%). Rural women (7%) were at a higher risk of hysterectomy compared with urban women (5%). A smaller proportion of women (5%) from the lowest wealth quintile had undergone hysterectomy compared with 7 % from the third and fourth quintiles. In percentage, more women (8%) with higher BMI (BMI > =25.0 kg/m^2^) were found to be at higher risk of undergoing hysterectomy compared with only around 6 % women with BMI < 25.0 kg/m^2^(Table 1).

Table 1 clearly shows that compared with women in the other category (3%), a higher proportion (7%) of widows had undergone hysterectomy, followed by currently married women (6%). The study finds that the percentage of hysterectomized women was high (10%) among women who had started cohabitation at a younger age, particularly below age 15 years. In terms of parity, the proportion of hysterectomized women increased with increase in the parity of a woman, standing at 7 % among women with third or higher order parity.

Table 1 also provides regional variations in the level of hysterectomy in India. The South (8%) and East (6%) regions show a prevalence of hysterectomy above the national level. The level for the North, Central and West regions turns out to be slightly lower than the national average (6%), whereas the least proportion of women undergoing hysterectomy was found in the Northeast region (2%).

Table 2 reveals the average number of years passed between when a woman underwent a hysterectomy and the survey date. Forty-six percent women had completed seven or more years since the operation had been done, followed by 31% who had passed less than 4 years. The rest of the 23% women had completed between four and 6 years.

**Table 2:** Time since hysterectomy and source where hysterectomy done among women age 30–49, India, NFHS (2015–16)

State/union territory	Number of women who undergone hysterectomy	Percentage distribution
Time since hysterectomy (in years)^a^		
0–3	6439	31.1
4–6	4750	23.0
7 and above	9476	45.9
Place of hysterectomy^b^		
Public health facilities	6687	32.2
Private health facilities	14,061	67.8
All	20,761	100.0

^a^96 women did not report timings of hysterectomy and ^b^13 women did not report source of hysterectomy in the survey

### Regional variations in type of health institutions where hysterectomy was sought

Women who had hysterectomies were further asked, “Where was this operation performed?” A little less than one-third (32%) of hysterectomies in India were done in public health facilities and the rest in private health facilities at the national level (Table 2). It is important to mention that the private health facilities also include a very small proportion (nearly 1%) of nongovernmental organizations (NGOs) or not-for-profit trusts.

The pattern in the north and the northeast regions was quite different from the country as a whole (data not shown). Sixty-one per cent of hysterectomies in the northeast region were done in public institutions, followed by 39% in the north. The utilisation of the public sector was the highest in Assam (64%), followed by Jammu and Kashmir (62%). None of the women in Lakshadweep had utilised public sector facilities. The utilisation of the private sector was more than 70% in Jharkhand (72%), Uttar Pradesh (76%), and Bihar (82%) [data not shown but can be provided on request].

### Results from multivariate logistic regression analysis

Table 3 shows the odds ratio (OR) from the multivariate logistic regression that was run to examine the likelihood of a woman undergoing hysterectomy (dependent variable) belonging to given sociodemographic background characteristics (independent variables). Woman’s age was found to be statistically associated with increase in the risk for hysterectomy. For example, women aged 45–49 were 3.8 times as likely as women aged 30–34 to have undergone a hysterectomy. Likewise, women aged 40–44 were 2.8 times more likely than women aged 30–34 to have undergone a hysterectomy. Woman’s schooling was negatively associated with hysterectomy. Women with more years of schooling were less likely to have undergone a hysterectomy compared with women with no schooling. For example, women with higher education were about 0.4 times less likely to have had a hysterectomy compared with women with no schooling. Women with “secondary complete” level of education were about 35% less likely to have had a hysterectomy compared with women with no schooling. Caste was also found to be associated with hysterectomy. Compared with women from scheduled castes, women from scheduled tribes were less likely to have had a hysterectomy. However, women from other backward classes and other castes were more likely to have undergone the operation than their scheduled caste counterparts. Muslim (OR:0.708, 95%CI[0.673–0.744]) and Buddhist/Neo-Buddhist (OR:0.499, 95%CI[0.400–0.622]) women were less likely to have had a hysterectomy compared with Hindu women. On the other hand, Sikh women (OR:1.173, 95%CI[1.035–1.331]) were more likely to have undergone a hysterectomy compared with Hindu women.

**Table 3:** Adjusted Odds Ratios obtained from the final step from multivariate logistic regression analysis

Background Characteristics		Categories	Exp(B)	95% CI for EXP(B)	
				Lower	Upper
Age		30–34			
		35–39	1.763^c^	1.675	1.855
		40–44	2.815^c^	2.680	2.957
		45–49	3.840^c^	3.658	4.031
Women’s Education		No schooling			
		Primary	.872^c^	.836	.909
		Secondary	.654^c^	.628	.681
		Higher	.447^c^	.410	.486
Caste/tribe		Scheduled caste			
		Schedule tribe	.896^b^	.839	.957
		Other backward class	1.252^c^	1.202	1.304
		Other	1.203^c^	1.148	1.260
Religion		Hindu			
		Muslim	.708^c^	.673	.744
		Christian	.980	.892	1.077
		Sikh	1.173^a^	1.035	1.331
		Buddhist/neo-Buddhist	.499^c^	.400	.622
		Others	.832	.674	1.028
Place of Residence		Urban			
		Rural	1.463^c^	1.411	1.518
Wealth Index		Lowest			
		Second	1.350^c^	1.281	1.423
		Middle	1.575^c^	1.492	1.662
		Fourth	1.780^c^	1.679	1.888
		Highest	2.029^c^	1.896	2.170
Body mass Index		BMI < 25 kg/m^2^			
		BMI < 25 kg/m^2^	1.392^c^	1.348	1.438
		BMI = not available	1.120^a^	1.014	1.236
Marital Status		Currently married			
		Widow	1.503	.726	3.114
		Others	1.196	.584	2.451
Starting Age at First Cohabitation		Less than 15			
		15–19	.665^c^	.641	.691
		20 and above	.459^c^	.438	.480
		Not applicable	.397^a^	.192	.820
Parity		None			
		Two-three	1.818^c^	1.632	2.026
		Three and above	1.850^c^	1.660	2.062
		North			
Region		Central	1.253^c^	1.179	1.332
		East	1.824^c^	1.715	1.939
		Northeast	.659^c^	.574	.756
		West	1.641***	1.540	1.748
		South	2.061^c^	1.946	2.183
		Constant	.031		

^a^5% level of significance; ^b^1% level of significance; ^c^0.1% level of significance

Women who lived in rural areas had an about 1.5 times greater likelihood of having had a hysterectomy as compared with women living in urban areas. Women from the higher wealth quintile households were found to be at a significantly higher risk for hysterectomy as compared with women from the lower wealth quintile households. For example, women from the highest quintile were 2.0 times more likely than women from the lowest quintile to have undergone a hysterectomy. Similarly, women from the second, third, and fourth quintiles were at a significantly higher risk of hysterectomy as compared to women from the lowest quintile.

Women’s body mass index was also found to be statistically significantly associated with the likelihood of having undergone a hysterectomy. Women with a BMI more than or equal to 25 kg/m^2^ were 1.4 times more prone to have undergone hysterectomy than their counterparts with a BMI less than 25 kg/m^2^. Age at first cohabitation (also known as age at consummation of marriage) predicted a negative and a significant association with the odds of having undergone a hysterectomy in the study population. Women who reported their age of first cohabitation at 20 years and above had 55% lower odds of having undergone hysterectomy than women who reported their first cohabitation at age less than 15 years.

In our analysis, women’s parity also came out as a significant predictor of hysterectomy. The study found that women of third and higher parity were 1.9 times more likely to have undergone a hysterectomy compared with nulliparous women. The odds of having had a hysterectomy were 82% higher for women who had either first or second parity compared with nulliparous women.

Women who lived in the South and East regions, respectively, were about 2.1 and 1.8 times as likely to have undergone a hysterectomy as women in the North region of India. Women who lived in the Central region were also about 1.3 times more likely to report a hysterectomy as compared with women in the North region. In contrast, women in the Northeast region were about 34% less likely to report having undergone a hysterectomy as compared with women from the North region.

The Omnibus tests shows that the model has significantly improved from the base to the full model at *p* < 0.000, and converged with Chi-square at the value of 11,410.59.

### Reasons for which hysterectomy was performed

The literature suggests that hysterectomy treats a number of conditions and diseases. These include chronic pain, excessive bleeding, endometriosis, pelvic floor prolapse, uterine and cervical cancers, uterine disorders, etc. The NFHS-4 asked all the women who had undergone a hysterectomy the question: “Why was this operation (hysterectomy) preformed?” It was a multiple response category question as there may be more than one reason for resorting to hysterectomy. Table 4 is generated by tabulating these responses from the dataset. The sample size (N) for each state and union territory is provided in the last column of the table.

Table 4 posits that the most frequently reported reason for undergoing hysterectomy at the national level was women suffering from excessive menstrual bleeding/pain (56%), followed by fibroid/cyst (20%). Slightly less than three-fourths of women (73%) in Chhattisgarh reported excessive menstrual bleeding/pain as one of the reasons for hysterectomy. Table 4 shows the percentage of women aged 30–49 who reported excessive menstrual bleeding and pain and fibroid/cysts, respectively, as reasons for hysterectomy in different states of India. Among the major states, like Maharashtra (61%), Karnataka (56%), Andhra Pradesh (55%), and Tamil Nadu (51%), more than 50% women had undergone hysterectomies because of excessive menstrual bleeding and pain. The percentage of women who had undergone a hysterectomy due to excessive bleeding and pain ranged between 25% and 50% in the remaining states of India.

**Table 4:** Percentage of women (30–49) undergone hysterectomy and reported various reasons for hysterectomy by State/UTs, NFHS(2015–16)

State/union territory	Excessive menstrual bleeding/Pain	Fibroids /Cysts	Uterine disorder (Rupture)	Cancer	Uterine prolapse	Severe post-partum haemorrhage	other	Total sample (N)
North								
Chandigarh	19.8	56.4	10.6	6.9	16.7	0.0	0.0	9
Delhi	44.9	21.1	22.7	5.4	3.5	2.1	6.2	115
Haryana	61.3	19.9	13.0	6.2	8.3	2.4	5.3	276
Himachal Pradesh	32.4	37.2	14.5	1.2	3.2	4.1	12.7	85
Jammu & Kashmir	43.6	36.9	19.3	1.3	7.9	3.1	4.5	170
Punjab	44.0	48.8	14.1	1.8	5.4	1.2	.9	397
Rajasthan	58.5	24.6	14.0	6.8	10.7	3.4	3.0	786
Uttarakhand	48.0	24.0	10.3	7.0	12.2	3.6	8.3	117
Central								
Chhattisgarh	73.1	15.7	11.7	3.4	5.6	2.5	4.4	299
Madhya Pradesh	61.8	17.3	13.6	5.5	6.5	3.6	7.8	1218
Uttar Pradesh	53.1	15.5	10.5	16.0	7.6	3.7	10.2	2251
East								
Bihar	54.6	9.1	18.0	7.0	18.1	5.5	5.8	2705
Jharkhand	54.4	15.7	19.2	4.8	9.8	7.2	7.1	371
Odisha	47.0	4.8	9.4	4.2	8.9	5.7	24.9	506
West Bengal	40.0	25.8	10.0	1.5	6.3	3.2	18.1	1102
Northeast								
Arunachal Pradesh	28.6	2.9	13.4	4.0	14.0	9.9	29.2	10
Assam	54.3	4.0	11.8	5.1	4.4	9.8	15.0	157
Manipur	39.4	30.6	18.8	2.5	11.1	5.4	4.6	19
Meghalaya	21.1	21.5	17.7	1.1	10.7	10.4	23.4	17
Mizoram	11.8	39.4	12.2	22.3	2.1	2.4	16.7	6
Nagaland	37.4	19.1	12.4	8.3	4.0	5.0	15.0	12
Sikkim	59.6	15.4	17.1	1.5	5.3	2.5	3.5	4
Tripura	34.9	1.5	14.6	2.7	4.7	14.0	32.0	27
West								
Dadra & Nagar Haveli	64.0	12.1	3.9	0.0	11.3	0.0	20.5	6
Daman & Diu	45.2	6.6	5.1	0.0	4.0	0.0	39.1	2
Goa	44.8	28.0	13.1	0.0	5.5	1.4	7.2	21
Gujarat	49.6	28.2	13.6	1.6	7.0	2.4	9.0	1339
Maharashtra	69.2	13.0	10.2	3.9	6.5	3.2	5.7	1654
South								
Andaman & Nicobar Islands	64.5	10.4	9.8	0.0	4.9	1.5	19.1	4
Andhra Pradesh	63.8	22.5	17.0	2.4	3.6	1.3	0.9	2556
Karnataka	64.4	19.2	10.0	3.6	5.3	3.8	4.1	968
Kerala	39.9	49.3	10.9	.7	5.9	3.0	1.0	349
Lakshadweep	–	–	–	–	–	–	–	1
Puducherry	78.1	26.2	2.5	1.8	2.9	0.0	1.4	13
Tamil Nadu	55.5	12.3	8.7	8.2	6.1	3.3	11.0	1622
Telangana	48.3	34.4	22.2	2.4	4.2	1.7	3.0	1567
India	55.6	19.8	13.9	5.5	7.8	3.4	7.1	20,761

Fibroids /cysts are the second self-reported indications of hysterectomy in India. The percentage of women who reported fibroids /cysts as the cause for their hysterectomies ranged from 2 % in Tripura to the highest of 49% each in Kerala and Punjab.

Himachal Pradesh (37%), Jammu and Kashmir (37%), and Telangana (34%) fell in the middle of this range (Table 4).

Nationally, 14% of women aged 30–49 years gave uterine rupture as one of the reasons for their hysterectomy, whereas about 8 % reported that uterine prolapse was one of the reasons for it. Women from Delhi in the North (23%) and Telangana (22%) in the South reported uterine rupture as the more frequent reason for hysterectomy than the national average.

It is to be noted that 6 % of women reported that cancer was also one of the reasons for hysterectomy at the national level. The proportion of women reporting cancer as a reason for hysterectomy was the highest among women in Uttar Pradesh (16%), Chhattisgarh (13%), and Tamil Nadu (8%). (Table 4).

Only 9 % of women who reported excessive menstrual bleeding/pan as one of the reasons for hysterectomy also reported suffering from fibroids/cysts before having undergone the surgery (Data not shown). On the other hand, of those women who reported fibroids/cysts as one of the reasons for hysterectomy, nearly a quarter (24%) reported that they had suffered from excessive bleeding too (Data not shown). Therefore, one can say that excessive menstrual bleeding/pain is not a coexisting condition for hysterectomy among Indian women.

## Discussion

This is the first study in India that provides the prevalence of hysterectomy among women aged 30–49 years from a nationally representative survey at the national, regional and state levels. It also provides social, economic and demographic determinants along with self-reported reasons for undergoing hysterectomy. This paper offers a comprehensive analysis regarding all these critical aspects of hysterectomy in the Indian context. In the western world, the prevalence of hysterectomy ranges from 10 to 20% among women in the latter half of the reproductive span. However, the findings of this study reveal that six in every 100 women aged 30–49 have had a hysterectomy in India. The prevalence is about 11 per 100 women in the age group 45–49 years. The study by Prusty, Choithani and Gupta (2018) also found hysterectomy prevalence ranging between 0.2 per to 6.3 per 100 women in the age group 15–49 years for 21 out of 36 states and UTs of India [8]. If one converts the prevalence among women aged 30–49 into absolute numbers, India will probably turn out to be the largest country in the world having women with such surgeries. Another worrisome factor is the low median age at hysterectomy in India. Our data reveals that the median age at hysterectomy is as low as 34 years at the national level and lower still among women belonging to the rural and lowest wealth quintile settings. This means half of the hysterectomies in India are done before women hit their mid-thirties. Similar results were obtained in a study by Desai et al. (2016), who estimated 36 years of median age at hysterectomy for women in a low income setting in the state of Gujarat [5]. This can have a huge impact on the women’s socio-psychological and physical health on account of the adverse effects of the long exposure of undergoing a hysterectomy at an early age [24].

Age is a significant and clear predictor for women undergoing hysterectomy. This may be attributable to more and more women being advised by doctors to undergo the surgery or to women themselves choosing to get rid of their health problems after achieving the desired number of children, all of whom have survived up to a certain age. Women with no schooling, those living in rural areas, those belonging to higher wealth quintiles, those with BMI > =25 kg/m^2^, those belonging to other castes, those of higher parity, those with a low age at first cohabitation, and those from eastern and southern regions are more likely to undergo hysterectomy. It is highly probable that women with no schooling and those from a rural background may have had a hysterectomy due to an infection or uterus-related morbidities. On the other hand, women belonging to well-off households (more likely from better off households and BMI > =25 kg/m^2^) may have had it because they were more likely to be able to afford the costly procedure of hysterectomy. Here it is important to mention that more than two-thirds (68%) of hysterectomies are reported to have been done in private health settings, where a woman’s family has to bear nearly the entire financial burden caused due to the procedure. The study by Desai, Sinha and Mahal [5] supports our finding that rural women are more likely to go for hysterectomies. The prevalence of hysterectomy is also somewhat linked with the low age at first birth and high number of births and untreated reproductive morbidities, as cited by Govil [4]. These are typical features of reproduction among rural women and women with no schooling in India. Generally, less educated women are less aware of reproductive health and hygiene [23]. Awareness about health check-ups and health-seeking behaviour are also low among uneducated women and those from poor socioeconomic backgrounds. These factors can potentially delay, or cause the women to avoid, seeking treatment at the initial stages of a reproductive health problem [25]. Most women do not seek treatment at the early stages of reproductive health problems because they perceive them to be normal for women. Medical treatments are sometimes regarded as an unnecessary intervention in the women’s reproductive health system [11].

The prevalence of hysterectomies in the states of Andhra Pradesh (16%), Telangana (14%), Bihar (11%), and Gujarat (8%) was found to be relatively higher than in the other states. The study by Prusty, Choithani and Gupta [8] too showed that Andhra Pradesh (6%), Telangana (5.5%), and Karnataka (3%) had a relatively higher prevalence than the remaining 18 states. They, however, found the prevalence in Andhra Pradesh and Telangana to be much lower than what our study has estimated. This discrepancy may be explained on the grounds that this study used data from the NFHS-4, which had developed a full battery of questions related to hysterectomy and had provided comprehensive training to field investigators on this medical procedure. This may have aided women in responding more clearly to the questions related to hysterectomy.

Telangana was carved out of Andhra Pradesh and, therefore, shares similar sociodemographic, cultural, and health practices. The NFHS-4 shows that about two-thirds of women in the reproductive age group in Andhra Pradesh and about 57% in Telangana were overweight or obese [23]. The fact that hysterectomy is associated with overweight and obesity and that women face early marriage and early initiation into childbearing in these two states could be why the prevalence of hysterectomy was found to be higher in these two states. Bihar is one of the most backward states in terms of socioeconomic development; generally, women there are less aware of reproductive health problems and the treatment options. It is also one of the least urbanised states of India. The higher prevalence of hysterectomy in Bihar is likely the outcome of poor access to public health infrastructure in rural areas in the state, the result being delay in treatment seeking for reproductive health problems and hysterectomy being used as the last resort to deal with them. Gujarat is one of the more developed states of India, where a high proportion of women belong to the high wealth quintile and where there is a good presence of the private health sector, which might partially lead to a high risk for hysterectomy. However, one should investigate whether the age group 40–49 is overrepresented since a high prevalence of hysterectomy was observed in this group, particularly in the state of Gujarat.

The leading self-reported causes of hysterectomy are excessive menstrual bleeding/pain (56%), followed by presence of fibroids/cysts (20%) and uterine ruptures (14%). Since the question had multiple answers, some of the reasons might be overlapping. However, most of the hospital based studies from other countries show that fibroids (73% in Hong Kong, 65% in India, 60% in USA, 33% in Pakistan, and 23% in South Africa), followed by prolapse remain the leading cause for hysterectomy [26–29]. A regional pattern emerged in this study – excessive menstrual bleeding or pain was the most common cause in Haryana, Madhya Pradesh, Chhattisgarh, Maharashtra, Karnataka, Andaman and Nicobar Islands, and Andhra Pradesh. On the other hand, fibroids or cysts were the most common cause of hysterectomy in Chandigarh, Punjab, and Kerala. Uttar Pradesh must draw the immediate attention of policy makers as cancer was the second most frequently reported reason for hysterectomy.

## Conclusion

This paper concludes that the likelihood of a woman undergoing hysterectomy increases with age, wealth quintile, and parity. On the other hand, it decreases with increasing years of schooling and age at first cohabitation. Sikh, rural, obese (BMI > =35 kg/m^2^) women, and those who reside in the south region possess a higher risk for hysterectomy than their counterparts in the respective reference groups. The other important contribution of this paper is providing reasons for hysterectomy from the population based nationally representative dataset. It concludes that the most frequent reason for undergoing hysterectomy was excessive menstrual bleeding/pain, followed by the presence of fibroid/cyst. There were large variations across the states and the UTs in reporting the reasons for undergoing hysterectomy. This paper also finds that the median age at hysterectomy is much lower among Indian women.

The conclusion clearly indicates the need to spread general awareness regarding reproductive health issues, treatment seeking, reducing high fertility, increasing age at first marriage, and cohabitation. Obese women should be advised to go for regular screening of reproductive health so that appropriate treatment other than hysterectomy can be tried first. More specific interventions are required to deal with the reproductive health problems reported by women as reasons for hysterectomy. Reproductive health program managers should devise state specific strategies to enable women to seek treatment at the initial stage so that unnecessary hysterectomies can be avoided. At the policy level, there is a pressing need to improve both the access to and the quality of reproductive health care services in public health facilities so as to reduce the growing dependency on hysterectomies in treating reproductive health problems. Specific intervention programs for the early screening, identification, and treatment of women’s sexual and reproductive health problems need to be formulated. The national health program on *women’s health and gender mainstreaming,* included in the recent National Health Policy [30], talks about enhanced provisions for reproductive morbidities and health needs of women beyond the reproductive age group (40+). However, neither this health policy nor the draft National Policy for Women [31] makes any mention of hysterectomy emerging as a public health issue. It is important, therefore, that the health and financial consequences of hysterectomy on women’s lives be addressed as part of comprehensive strategies for the screening, necessity and after-effects of hysterectomy while designing the national health program for women. The policy documents should also expand the intended age group since many hysterectomies are performed on women below the age of 40 years. There is a need to devise strategies to deal with the burden of unnecessary hysterectomies by developing some regulatory or ethical clearance mechanism especially for private sector providers. These concerns have also been reported by many studies in the past [18, 19, 32, 33]. In addition to causing multiple health consequences, hysterectomy may also lead to catastrophic health expenditure and may push a family into poverty due to heavy dependence on the private health sector, especially it is not protected under any health insurance scheme. Even when a family avails treatment in the public sector, women are likely to face financial and health implications.

The policy makers need to be cautioned about the probable scenario where the number of unnecessary hysterectomies may go up because of the availability of laparoscopic technology [34] and cashless protection under the *Ayushman Bharat* health program [35]. Women may avoid hysterectomy because of the fear of open surgery that requires a longer hospitalization and healing time or because of the lack of financial resources. But access to the new laparoscopic hysterectomy that it is being promoted as a minimal or non-invasive procedure and that requires a much shorter hospital stay or healing time may mitigate those fears. The entitlements under the *Ayushman Bharat* scheme may also be misused in case of women from the poor sociodemographic strata. Therefore, implementing authorities must ensure some kind of regulatory mechanism while making payments to private providers for these women.

### Limitations of the study

The present study has limitations since the NFHS-4 does not provide data on the costs incurred or the financial loss faced by women or their families due to hysterectomy. We suggest that the future rounds of NFHS collect such important policy-relevant information from each woman who has undergone a hysterectomy. Data should also be collected on its different aspects like whose decision it was to go for the surgery, for how long the woman had been suffering from health problems due to which hysterectomy had to be done, where and for how long the woman had been undergoing treatment before she resorted to the operation, and what the sources of finance were. The NFHS-4 dataset does not provide information as to whether a woman was operated through the laparoscopic technique or through the usual surgical procedure, or whether it was abdominal or vaginal, or whether a woman had any side effect of hysterectomy. It also does not provide any information on whether the hysterectomy was done after all other treatment methods had been exhausted.

As for the reasons of hysterectomy, no interpretations could be made for the state of Goa, for UTs other than Delhi, and for Northeast states except Assam and Tripura due to the small sample size (less than 25 cases). The future NFHS round may consider larger sample of women from these states and UTs. The above information would be extremely useful in developing strategies for quality service provisions to those women who genuinely need to undergo hysterectomy in India.

## Data Availability

The data for this research is available to the public on DHS measures website. Any individual can register and easily obtained data in electronic version from the following website https://dhsprogram.com/data/new-user-registration.cfm

## Abbreviations

BMI: Body mass index
CEBs: Census enumeration blocks
CI: Confidence interval
GIS: Geographical information system
HPE: Histopathological examination
IIPS: International institute for population sciences
IUDs: Intrauterine devices
NFHS: National family health survey
NGOs: Nongovernmental organization
OR: Odds ratio
PPS: Probability proportion to size
SPSS: Statistical package for social sciences
UTs: Union territories

## Footnotes

1 North (Chandigarh, Delhi, Haryana, Himachal Pradesh, Jammu & Kashmir, Punjab, Rajasthan, Uttarakhand); Central (Chhattisgarh, Madhya Pradesh, and Uttar Pradesh); East (Bihar, Jharkhand, Odisha, West Bengal); Northeast (Arunachal Pradesh; Assam, Manipur, Meghalaya, Mizoram, Nagaland, Sikkim, Tripura); West (Dadra & Nagar Haveli, Daman & Diu, Goa, Gujarat, Maharashtra); South (Andaman & Nicobar Island, Andhra Pradesh, Karnataka, Kerala, Lakshadweep, Puducherry, Tamil Nadu, Telangana)
